# Carbon metabolism modulates the efficacy of drugs targeting the cytochrome *bc*_1_*:aa*_3_ in *Mycobacterium tuberculosis*

**DOI:** 10.1038/s41598-019-44887-9

**Published:** 2019-06-13

**Authors:** Nitin P. Kalia, Bei Shi Lee, Nurlilah B. Ab Rahman, Garrett C. Moraski, Marvin J. Miller, Kevin Pethe

**Affiliations:** 1 Lee Kong Chian School of Medicine, Nanyang Technological University, Singapore, 636921 Singapore; 2 School of Biological Sciences, Nanyang Technological University, Singapore, 637551 Singapore; 3 Department of Chemistry and Biochemistry, Montana State University, Bozeman, MT 59717 USA; 4 Department of Chemistry and Biochemistry, University of Notre Dame, Notre Dame, IN 46556 USA

**Keywords:** Tuberculosis, Antimicrobials

## Abstract

The influence of carbon metabolism on oxidative phosphorylation is poorly understood in mycobacteria. *M*. *tuberculosis* expresses two respiratory terminal oxidases, the cytochrome *bc*_1_*:aa*_3_ and the cytochrome *bd* oxidase, which are jointly required for oxidative phosphorylation and mycobacterial viability. The essentiality of the cytochrome *bc*_1_*:aa*_3_ for optimum growth is illustrated by its vulnerability to chemical inhibition by the clinical drug candidate Q203 and several other chemical series. The cytochrome *bd* oxidase is not strictly essential for growth but is required to maintain bioenergetics when the function of the cytochrome *bc*_1_*:aa*_3_ is compromised. In this study, we observed that the potency of drugs targeting the cytochrome *bc*_1_*:aa*_3_ is influenced by carbon metabolism. The efficacy of Q203 and related derivatives was alleviated by glycerol supplementation. The negative effect of glycerol supplementation on Q203 potency correlated with an upregulation of the cytochrome *bd* oxidase-encoding *cydABDC* operon. Upon deletion of *cydAB*, the detrimental effect of glycerol on the potency of Q203 was abrogated. The same phenomenon was also observed in recent clinical isolates, but to a lesser extent compared to the laboratory-adapted strain H37Rv. This study reinforces the importance of optimizing *in vitro* culture conditions for drug evaluation in mycobacteria, a factor which appeared to be particularly essential for drugs targeting the cytochrome *bc*_1_*:aa*_3_ terminal oxidase.

## Introduction

*Mycobacterium tuberculosis* is a heterotrophic bacterium capable of deriving energy from a wide array of carbon sources when grown in artificial culture broth media^1–5^. Yet, constraints imposed by the host restrict access to certain nutrients, forcing *M*. *tuberculosis* to use a limited pool of carbon sources as energy supplies, mainly in the form of lipids, and probably lactate^6–8^. While the glycolytic pathway is dispensable for virulence in animals^9^, gluconeogenesis is required for growth and persistence^10^. Remarkably, mycobacteria deficient for gluconeogenesis are unable to establish infection and are cleared from the host at a very fast rate^5,10^. Nevertheless, mycobacteria are still able to scavenge a limited amount of host-derived glucose used primarily as a precursor for biosynthetic pathway rather than for energy production^11^. It is interesting to note that the absence of catabolic repression in mycobacteria allow the bacteria to co-metabolize several carbon sources^12^, a property that may confer a survival advantage in an environment in which resources are scarce such as in the lung granuloma^13^. NAD^+^ is a crucial cofactor involved in redox cellular balance, catabolism and energy production^14^. When carbohydrates are provided as carbon and energy source, NAD^+^ is reduced to NADH by glycolytic enzymes, which is in turn re-oxidized back either by the respiratory NADH dehydrogenases, or by fermentative enzymes^15^. Carbon and energy metabolism are tightly regulated in bacteria. Even though ATP is the central energy currency in all bacteria, strategies to resynthesize it can differ significantly from one bacterium to the next. Many bacteria including enterobacteria are able to bypass the essentiality of the oxidative phosphorylation pathway when grown on carbohydrates, doing so by regenerating the NAD^+^ pool by fermentation^16,17^. Mycobacteria are unable to do so either in culture broth media or in animal models. The absence of fermentative (NADH-dependent) lactate dehydrogenase in *M*. *tuberculosis* makes the oxidative phosphorylation pathway strictly essential for growth^18^. This is illustrated by the successful clinical development of bedaquiline (Sirturo^®^), a drug targeting the F_O_F_1_ ATP synthase^19–21^. Since drugs targeting the Electron Transport Chain are effective against phenotypic drug-resistant mycobacteria^22^, they could be of value to shorten TB treatment time. In addition of the F_O_F_1_ ATP synthase, type II NADH dehydrogenases^23,24^, the menaquinone biosynthetic pathway^25,26^, and the cytochrome *bc*_1_*:aa*_3_ terminal oxidase^27–29^ are chemically-validated drug targets in *M*. *tuberculosis*. The qcrB subunit of the cytochrome *bc*_1_*:aa*_3_ is the target of the clinical drug candidate Q203^29^, as well as numerous other chemical series^27–29^. Despite a central role in metabolism and host adaptation, it is still largely unknown how carbon catabolism is coupled to oxidative phosphorylation in mycobacteria. Here we show that the potency of drugs targeting the cytochrome *bc*_1_*:aa*_3_ are modulated by carbon catabolism and the composition of the culture broth medium. Glycerol supplementation in the widely used 7H9 broth medium had a detrimental effect on the potency of the imidazopyridine carboxamide Q203 and ND-10885^30^, another *in vivo* active imidazopyridine carboxamide under study. This phenomenon was explained by a significant up-regulation of the Cyt*-bd* terminal oxidase that diminishes the potency of cytochrome *bc*_1_*:aa*_3_ inhibitors by providing an alternate respiratory route.

## Results and Discussion

### Glycerol supplementation interferes with the potency of drugs targeting the Cyt*-bc*_1_*:aa*_3_ in mycobacteria

**Figure 1:**
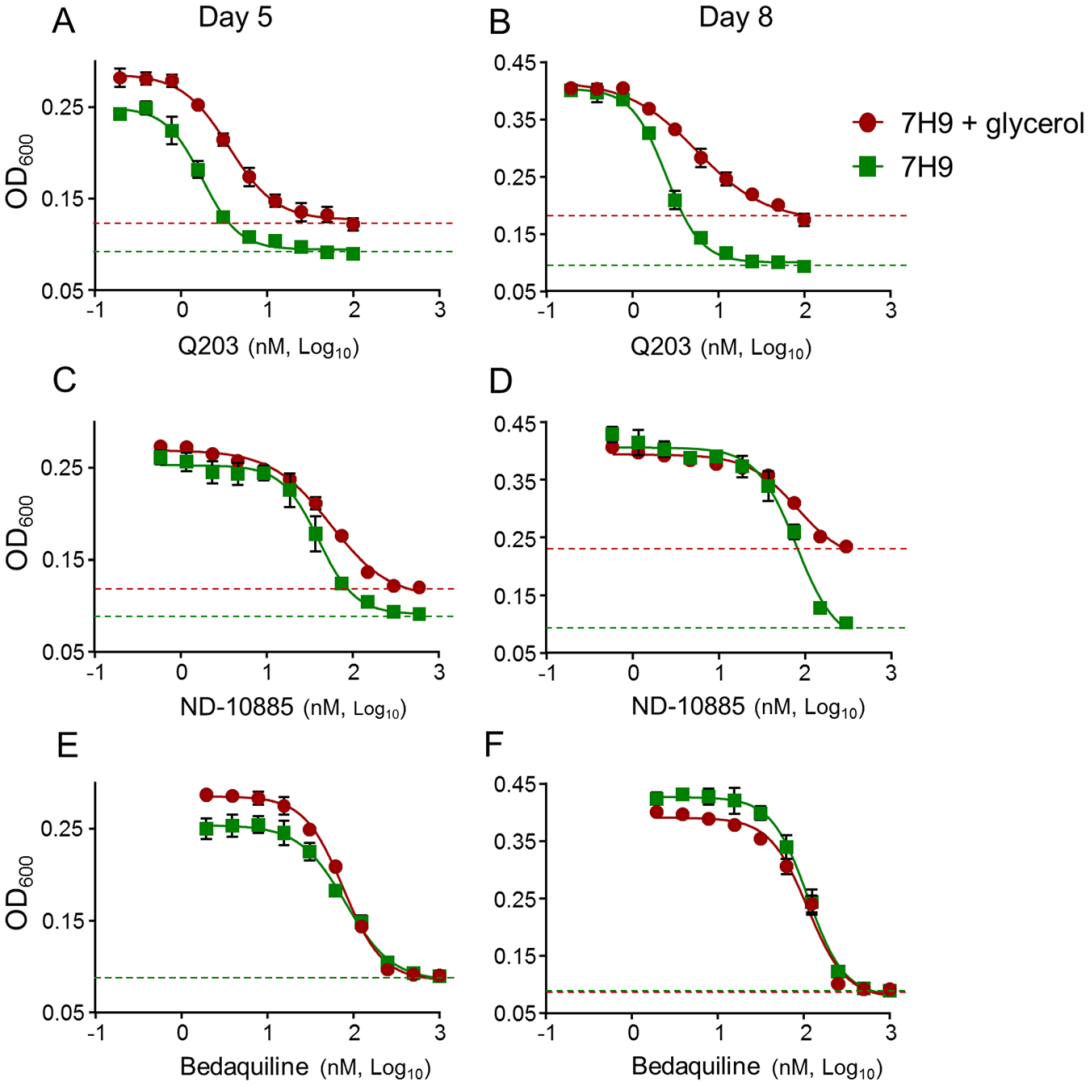
Potency of drugs targeting the Cyt*-bc*_1_*:aa*_3_ is influenced by carbon metabolism. MIC_50_ of Q203, ND-010885 and bedaquiline against *M*. *tuberculosis* H37Rv after 5 days (**A**,**C**,**E**) or 8 days (**B**,**D**,**F**) of incubation in the presence (red circles) or absence (green squares) of glycerol. Data are expressed as the mean ± SDs of triplicates for each concentration. Green dotted line: baseline for the dose-response curve without glycerol; red dotted line: baseline for the dose-response curve with glycerol.

Despite the discovery of multiple chemical classes targeting the cytochrome *bc*_1_*:aa*_3_, there are some conflicting reports on the consequences of chemical inhibition of the target on growth inhibition under laboratory conditions^31–33^, as well as reported difficulties to isolate escape mutants^34^. We hypothesised that conditions used to test drug potency may influence apparent activity. Since glycerol supplementation in 7H9-based medium is one of the parameters that varies widely between laboratories, and a factor known to influence drug potency^35^, we tested the efficacy of Q203 in 7H9 culture broth medium with and without glycerol in a turbidity-based assay. The Minimum Inhibitory Concentration inhibiting 50% of the growth (MIC_50_) were comparable after 5 or 8 days of incubation. However, maximum growth inhibition was not achieved in the presence of glycerol (Fig. 1A,B), suggesting that Q203 treatment was growth-retarding but not bacteriostatic in glycerol-supplemented medium. This phenomenon was also observed with ND-10885, a preclinical drug candidate targeting the cytochrome *bc*_1_*:aa*_3_^30^ (Fig. 1C,D). In comparison, the maximum growth inhibition of bedaquiline was not affected by the presence of glycerol (Fig. 1E,F).

**Figure 2:**
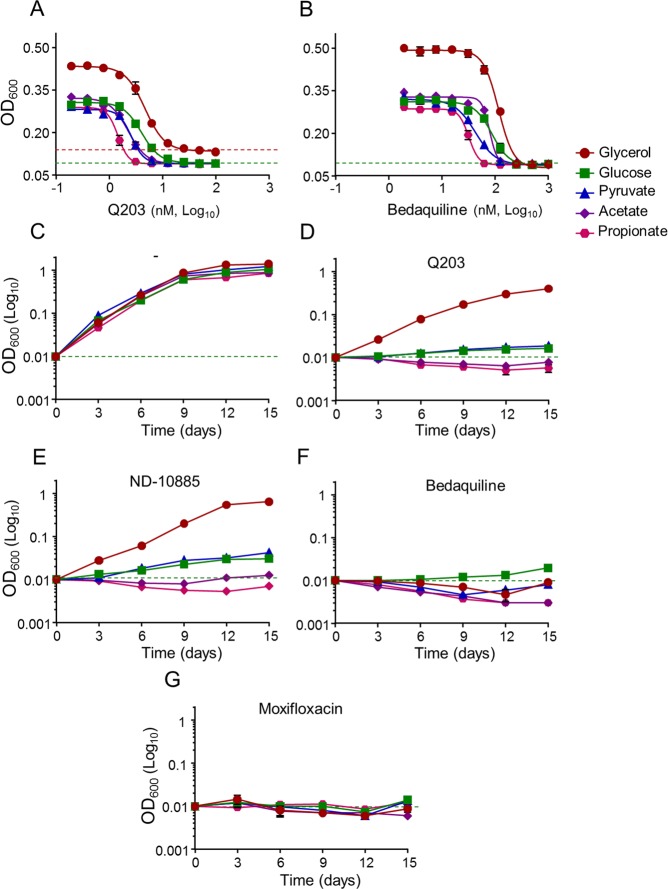
QcrB inhibitors are less than bacteriostatic against *M*. *tuberculosis* H37Rv on glycerol-supplemented medium. Potency of Q203 (**A**) and bedaquiline (**B**) against *M*. *tuberculosis* H37Rv growing in defined culture broth media supplemented with glycerol (red circles), glucose (green squares), pyruvate (blue triangles), acetate (purple diamonds) and propionate (pink hexagon) as sole carbon sources. MIC_50_ were recorded after 10 days of incubation. (**D**–**G**) *M*. *tuberculosis* H37Rv was inoculated on defined culture broth media supplemented with glycerol (red circles), glucose (green squares), pyruvate (blue triangles), acetate (purple diamonds) and propionate (pink hexagon) as sole carbon sources without drugs (**C**), 100 nM of Q203 (**D**), 2000 nM of ND-10885 (**E**) 500 nM of bedaquiline (**F**), or at 1,000 nM of moxifloxacin (**G**). Bacterial growth was monitored over a 15 days period. Error bars represent the standard deviation (SD) of three biological replicates from a single experiment.

**Figure 3:**
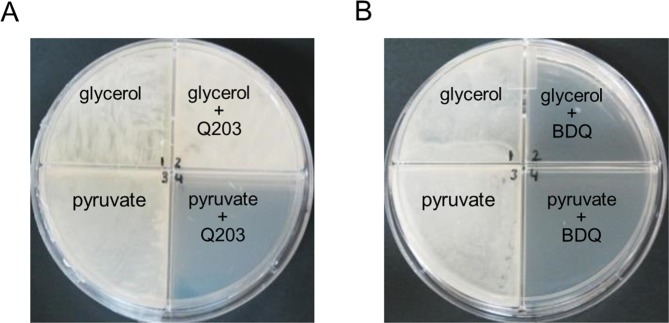
Glycerol supplementation suppresses the potency of Q203 on agar plates. *M*. *tuberculosis* H37Rv was plated on agar plates at a density of 1 × 10^6^ bacilli on 7H10-OADC plates supplemented with glycerol (0.5%) or pyruvate (20 mM) and containing no antibiotics (A, B: left quadrants), 100 nM of Q203 (**A**, right quadrants), or 500 nM of BDQ (**B**, right quadrants). Pictures were taken after 15 days of incubation at 37 °C.

Since the classical 7H9-ADS-tween 80 medium provides several potential energy and carbon sources, we profiled the potency of Q203 in a home-made 7H9-base medium containing either glycerol, glucose, pyruvate, acetate or propionate as dominant carbon sources. As a control, we showed that mycobacteria multiplied very minimally on the base medium without any dominant carbon sources (Fig. S1). Results confirmed that glycerol metabolism has a detrimental effect on the potency of Q203, both in *M*. *tuberculosis* H37Rv and in *M*. *bovis* BCG. Indeed, the drug candidate was unable to inhibit mycobacterial growth, as witnessed by the high value of the bottom plateau on glycerol (Figs 2A and S2A). We also observed that the MIC_50_ value of Q203 was the highest in glycerol medium and the lowest on propionate and acetate medium (Table S1). On all carbon sources except glycerol, Q203 and ND-10885 inhibited mycobacterial growth as well as bedaquiline or moxifloxacin (Fig. 2D–G). Quantification of extracellular glycerol concentration over a period of 12 days revealed that Q203-treated mycobacteria consumed glycerol efficiently, confirming the absence of growth inhibition of the drug candidate on this carbon source (Fig. S3A). Incidentally, we also observed that the MIC_50_ values of bedaquiline varied as a function of the carbon source, but maximum growth inhibition was achieved in all culture broth media (Fig. 2B, Table S1). The detrimental effect of glycerol supplementation on the potency of Cyt*-bc*_1_*:aa*_3_ inhibitors was also apparent on 7H10 agar plates. Q203 at 100 nM was unable to inhibit mycobacterial growth on 7H10 agar plates supplemented with glycerol after 18 days of incubation, whereas it was potent on 7H10 agar plates supplemented with pyruvate (Fig. 3A).

### Q203 treatment in glycerol-supplemented medium induces the overexpression of the *cydABDC* operon

Given the synthetic lethal interaction between the terminal oxidases in mycobacteria^31,36^, we hypothesized that overexpression of either of the terminal oxidases could explain the glycerol phenomenon. Therefore, we monitored the expression of the *qcrCAB* and *cydABDC* operon in mycobacteria growing in 7H9 medium with or without glycerol. After overnight culture in broth medium without glycerol, *M*. *bovis* BCG was transferred to fresh 7H9 medium with or without 0.2% glycerol supplement, and exposed to 100 nM of Q203 or DMSO control. After 72 hours of incubation, RNAs were extracted and the relative abundance of the terminal oxidases-encoding genes were evaluated by quantitative RT-PCR. Results revealed that glycerol supplementation, or Q203 treatment, had limited effect on the expression of the *qcrCAB* operon (Table 1). Conversely, glycerol supplementation, or Q203 treatment, triggered a 2- to 3-fold increase in expression of the *cydABDC* operon, whereas the combination of both stimulated a 7- to 8-fold induction (Table 1). These results suggested that the induction of the Cyt*-bd* expression on glycerol-supplemented 7H9 medium provided an efficient alternate pathway for the electron transport chain, thereby diminishing the potency of Q203 and related drugs. This idea was supported by the finding that over-expression of the *cydABDC* operon on a multi-copy plasmid partially diminished partially the growth inhibition potency of Q203 on acetate-supplemented 7H9 medium (Fig. S4).

**Table 1:** Glycerol and Q203 treatment triggers an upregulation of the *cydAB* operon in mycobacteria.

Genes	Expression level (Fold change)			
	−Glycerol		+Glycerol	
	−	+Q203	−	+Q203
*cydA*	1.0	2.7 ± 0.7	**2**.**2** ± **0**.**1**	8.1 ± 0.2
*cydB*	1.0	2.9 ± 0.4	**2**.**2** ± **0**.**2**	7.4 ± 0.3
*qcrC*	1.0	0.8 ± 0.4	0.5 ± 0.1	1.4 ± 0.4
*qcrA*	1.0	0.75 ± 0.4	0.47 ± 0.1	1.01 ± 0.5
*qcrB*	1.0	0.90 ± 0.3	0.50 ± 0.1	1.31 ± 0.6

Expression levels were measured by qRT-PCR. The gene *sigA* was used as an endogenous control (housekeeping gene) to determine the relative expression of the *M*. *bovis qcrCAB* and *cydAB* operons in culture broth media supplemented with glycerol and/or Q203.

**Figure 4:**
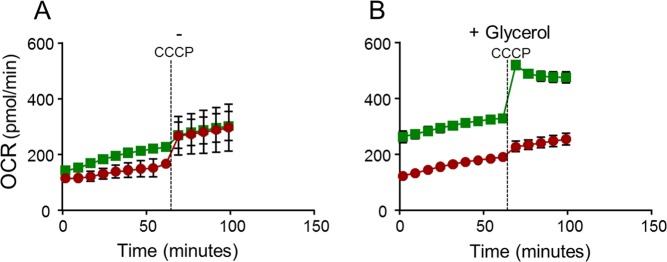
Q203 treatment increases the Oxygen Consumption Rate (OCR) in mycobacteria on glycerol supplemented medium. *M*. *bovis* (BCG) was incubated in 7H9 medium in absence (**A**) and presence (**B**) 0.2% glycerol. Mycobacteria were treated without (red circles) or with (green squares) 100 nM Q203 for 48 hours before recording the basal Oxygen consumption with a Seahorse XFe96 analyzer. CCCP was injected at 60 min post-recording (dotted line). Each reading was performed in triplicate and results expressed as mean ± SD.

It was also observed that the mycobacteria basal Oxygen Consumption Rate (OCR) on glycerol supplemented medium was significantly increased after treatment with Q203 for 48 hours (Fig. 4A,B). This result suggests that chemical inhibition of the Cyt-*bc*_1_*:aa*_3_ terminal oxidase stimulates respiration through the Cyt-*bd* branch.

### The carbon source-dependency of the Cyt*-bc*_1_*:aa*_3_ inhibitors is alleviated upon *cydAB* deletion

**Figure 5:**
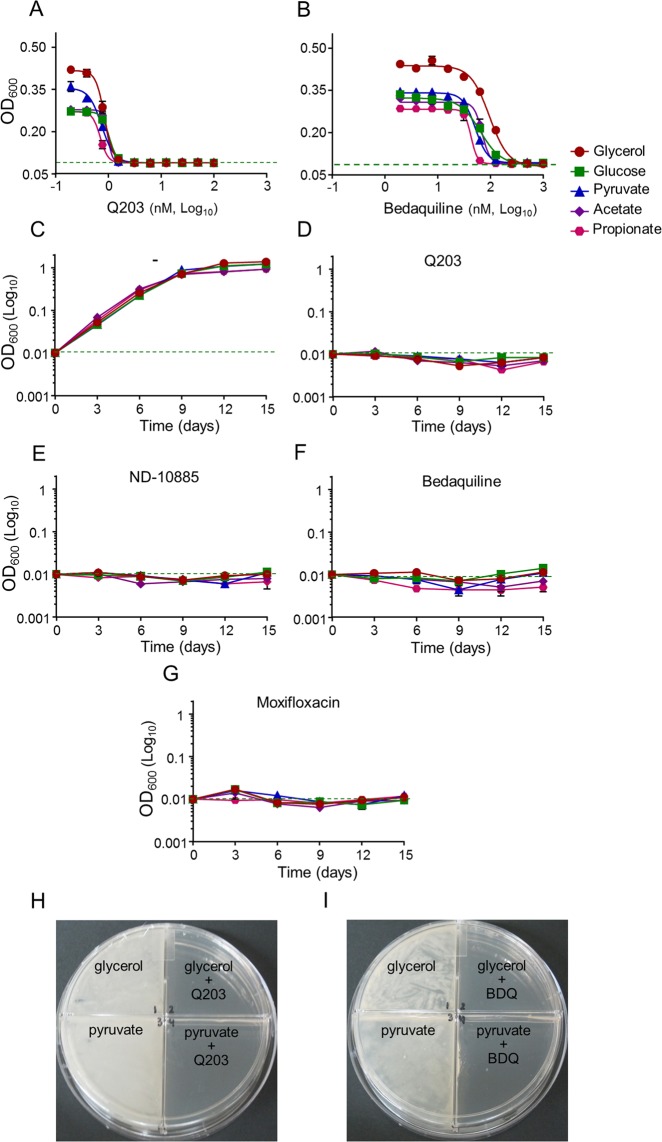
Potency of drugs targeting the Cyt*-bc*_1_*:aa*_3_ is carbon-source independent in the absence of *cydAB*. *M*. *tuberculosis* H37RvΔ*cydAB* was incubated with a dose-range of Q203 (**A**) and bedaquiline (**B**) in defined liquid broth media supplemented with glycerol (red circles), glucose (green squares), pyruvate (blue triangles), acetate (purple diamonds) and propionate (pink hexagon) as sole carbon sources for 10 days. Bacterial growth was measured by recording the optical density at 600 nm. Liquid broth media supplemented with the same carbon sources were also used to compare the growth of H37RvΔ*cydAB* in drug free conditions (**C**) with 100 nM of Q203 (**D**), 2000 nM of ND-10885 (**E**), 500 nM of bedaquiline (**F**) or 1,000 nM of moxifloxacin (**G**). *M*. *tuberculosis* H37RvΔ*cydAB* was plated on agar plates at a density of 1 × 10^6^ bacilli on 7H10-OADC plates supplemented with glycerol (0.5%) or pyruvate (20 mM) and containing no antibiotics (H, I: left quadrants), 100 nM of Q203 (**H**, right quadrants), or 500 nM of BDQ (**I**, right quadrants). Pictures were taken after 15 days of incubation at 37 °C.

To verify this assumption, the potency of Q203 was tested against the strains *M*. *tuberculosis* H37RvΔ*cydAB* and BCGΔ*cydAB* that do not express Cyt-*bd*^36^. Results revealed that maximum growth inhibition was achieved against the mutant strain, irrespective of glycerol supplementation (Fig. S5A). Reintroduction of a functional *cydAB* copy in the strain H37RvΔ*cydABcomp*^36^ restored the phenotype (Fig. S5C). These results were extended to defined culture broth media containing unique dominant carbon sources. Results confirmed that the potency of Q203 and the related drug ND-10885 was not influenced by carbon metabolism in the H37Rv Δ*cydAB* strain (Fig. 5D,E). In the absence of a functional Cyt*-bd*, the MIC_50_ of Q203 was shifted toward the lowest values previously obtained against the parental strains replicating on propionate (Figs 5A, S2C, Table S2). In comparison, the MIC values of bedaquiline were not affected by the absence of a functional Cyt-*bd* (Figs 5B, S2D, Table S2). Furthermore, growth of the H37Rv Δ*cydAB* strain was completely inhibited by Q203, even when glycerol was provided as sole carbon source (Fig. 5D), and to the same extent observed with bedaquiline (Fig. 4F) or moxifloxacin (Fig. 5G). These results demonstrated that the electron flow to the mycobacterial respiratory oxidases is modulated by carbon metabolism and influences the potency of the clinical candidate Q203. When tested on glycerol-supplemented agar plates, Q203 inhibited the growth of the H37Rv Δ*cydAB* strain as efficiently as bedaquiline (Fig. 5H).

### Glycerol metabolism interferes with the selection of spontaneous resistant mutants to Q203

We reasoned that the detrimental effect of glycerol metabolism may be the reason behind the reported difficulty to select escape mutants resistant to *qcrB* inhibitors^34^. In our own experience, spontaneous mutants resistant to Q203 could only be obtained at very high drug concentration^29^ and picked between day 12 and 14, before a lawn would appear on the plates (unpublished observation). To test this hypothesis, selection of escape mutants resistant to Q203 was attempted in the BCG parental and Δ*cydAB* strains on glycerol- or pyruvate-supplemented agar plates. 3 × 10^8^ bacilli were plated on agar plates containing Q203 at 500 nM and 100 nM. Visual examination of the plates after eighteen days of incubation at 37 °C revealed a thick lawn when the parental strain was plated on the glycerol plates (Table S3). Conversely, individual colonies were observed on the plates containing Q203 on pyruvate-supplemented plates (Table S3), showing that escape mutants to Cyt*-bc*_1_*:aa*_3_ inhibitors is facilitated by the omission of glycerol supplementation. Among all conditions tested, the easiest way to select spontaneous-resistant mutants was in the Δ*cydAB* background. Indeed, in the absence of a functional Cyt*-bd*, escape mutants to Q203 could be obtained at a frequency in the range 4.3 × 10^−8^ to 8.7 × 10^−8^, at a drug concentration as low as 5 nM, irrespective of the presence of glycerol (Table S3). Resistance to Q203 correlated with a single-nucleotide polymorphism in *qcrB* in all the mutants that were analysed (Table S3).

### The potency of Q203 is less affected by glycerol supplementation in *M*. *tuberculosis* clinical isolates

**Figure 6:**
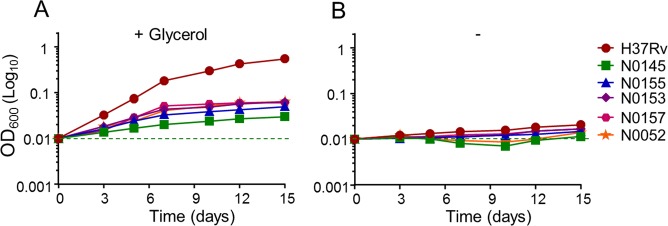
The potency of Cyt*-bc*_1_*:aa*_3_ inhibitors is less affected by glycerol supplementation in recent clinical isolates. The laboratory strain *M*. *tuberculosis* H37Rv (red circles), and the recent clinical isolates N0145 (green squares), N0155 (blue triangles), N0153 (purple diamonds), N0157 (pink hexagon) and N0052 (orange stars) were grown in the presence of Q203 at 50X its MIC_50_ in 7h9 liquid broth medium supplemented (**A**) or not (**B**) with 0.2% glycerol. Bacterial growth was recorded over a 15 days period by following the optical density at 600 nm. Each reading was performed in triplicate and results expressed as mean ± SD.

Lastly, we were interested in testing if the negative influence of glycerol supplementation was restricted to the laboratory-adapted *M*. *tuberculosis* strain H37Rv, or could be extended to recent clinical isolates. The growth kinetics presented on Fig. 2D was repeated in the clinical strains N0145, N0155, N0153, N0157 and N0052^37–39^. We noted that the glycerol effect was still noticeable on the clinical isolates, but the phenotype was very less pronounced compared to H37Rv (Fig. 6). This result points to an abnormal deregulation of the *cydABDC* operon in *M*. *tuberculosis* H37Rv, as reported previously^31^, and in *M*. *bovis* BCG. RT-PCR analysis confirmed a reduced expression of the *cydABDC* operon in the clinical isolates used in this study compared to H37Rv (Table 2).

**Table 2:** Relative expression levels of *cydA* and *cydB* in *M*. *tuberculosis* clinical isolates.

Clinical isolates #	Fold change in expression relative to H37Rv	
	*cydA*	*cydB*
N0153	0.64 ± 0.02	0.63 ± 0.02
N0157	0.59 ± 0.05	0.58 ± 0.04
N0052	0.35 ± 0.08	0.41 ± 0.1
N0155	0.31 ± 0.02	0.32 ± 0.02
N0145	0.15 ± 0.01	0.16 ± 0.02

RNA was extracted from early log-phase standing cultures growing in 7h9 liquid broth medium. Gene expression was normalized relative to H37Rv, *sigA* gene was used as an endogenous control.

Several lessons can be drawn from this study. It is first a reminder that despite its attractiveness, drug development against bacterial central metabolism and energetics targets is complex since the essentiality of those targets may be conditional and influenced by multiple factors such as the composition of the culture broth medium^40^. Developing screening conditions that reflect at least some of the conditions encountered by the bacteria during infection is primordial. Since glycerol metabolism does not seem to be relevant during infection^35^, we suggest that it should be omitted from the 7H9-based medium when screening or developing novel anti-TB drugs, which is particularly important for drugs targeting oxidative phosphorylation. The study also highlights possible biases that may be introduced by using laboratory strains expressing abnormal Cyt*-bd* level. In the quest of developing a drug combination targeting both terminal oxidases, we suggest that recent clinical isolates should be used alongside the laboratory-adapted strain H37Rv to guide chemical optimization and mode of action studies of drugs targeting the Cyt-*bc*_1_*:aa*_3_. Furthermore, the observation made in *M*. *tuberculosis* H37Rv suggests that a deregulation of the Cyt*-bd* expression could be a mechanism triggering resistance to drugs targeting the Cyt*-bc*_1_*:aa*_3_. This is a consideration that should be kept in mind as Q203 is entering into clinical phase 2 proof of concept studies^41^.

## Materials and Methods

### Strains and growth conditions

*M*. *tuberculosis* and derivative strains were maintained in Middlebrook 7H9 broth medium supplemented with 0.2% glycerol, 0.05% Tween 80, and 10% Album-Dextrose-Saline (ADS) supplement. Hygromycin (80 μg/mL) was used when required. The defined medium was based on 7H9 medium with the omission of glucose, tween 80 (replaced by Tyloxapol 0.05%) and BSA fraction V (substituted by 0.1% fatty-acid free BSA). All carbon sources were used at 0.2% except propionate and pyruvate which were used at 0.1% and 20 mM respectively. Q203 and bedaquiline (BDQ) were custom made at GVK Bio, moxifloxacin was purchased from Sigma and ND-10885 was synthesized as described before^30^.

All the activities pertaining to *M*. *tuberculosis* were performed in a registered Biosafety Level 3 facility, whereas the work on *M*. *bovis* (BCG) was carried out in a licensed Biosafety Level 2 facility.

### MIC_50_ determination

MIC_50_ were determined as previously described, with slight modifications. Briefly, drugs dissolved in 90% DMSO were twofold serial-diluted in duplicates and spotted to 96-well flat bottom plates. A volume of 200 µl of *M*. *tuberculosis* H37Rv culture (OD_600_ 0.005) was dispensed into 96 well flat bottom plates and the assay plates were incubated at 37 °C. Growth was determined by recording absorbance at 600 nm (OD_600_) on BioTek Cytation multimode reader after 5, 8 and 10 days of incubation. Absorbance values (OD_600_) were plotted using GraphPad Prism version 6 software and MIC_50_ were calculated from the curves obtained^35,36^.

### Growth inhibition assays on agar plates

Growth of *M*. *tuberculosis* was compared using a quadrant plate in presence and absence of Q203 on Middlebrook 7H10-OADC agar plates supplemented either with glycerol (0.5%) or pyruvate (20 mM). In brief, 50 µl of a suspension of 10^6^ mycobacteria was plated on each quadrant plates. Mycobacterial growth was observed after two to three weeks of incubation at 37 °C^42^.

### Growth kinetic assays

*M*. *tuberculosis* was first grown to mid-log phase in 7H9 liquid media supplemented with 10% ADS supplement, 0.2% glycerol and 0.05% of tween 80, washed twice in 7H9 base medium supplemented with 0.05% tyloxapol, without carbon sources, and re-suspended in the same medium. The cells were inoculated at an initial OD_600_ of 0.005 in 7H9 liquid broth medium supplemented with 0.1% fatty acid free BSA, 0.8% NaCl, and glucose, glycerol, pyruvate, acetate, or propionate as sole carbon source at the concentrations mentioned above. Bacterial growth was monitored at OD_600_ over time using an Eppendorf’s Biophotometer plus spectrophotometer.

### RT-PCR

Cells from mid-log phase culture of *M*. *bovis* (BCG) were washed and inoculated at an initial OD_600_ of 0.2 in 7H9 liquid media (10% ADS and 0.05% of tween 80) supplemented with and without 0.2% glycerol. Bacterial cultures in their respective media were incubated at 37 °C for 72 hrs in presence and absence of Q203. Total RNA was extracted using Max Bacterial Enhancement Reagent and TRIZOL^®^ according to manufacturer’s instruction (Ambion). Cell lysis was achieved by vigorous vortexing for 10 minutes using Disruptor Genie (Scientific Industries), following by DNase (TURBO DNA-free kit, Ambion) treatment. RNA concentrations were determined with a NanoDrop 2000c spectrophotometer.

First strand cDNA was synthesized from 1 µg of RNA for each sample using qScript cDNA Supermix (Quanta Biosciences). cDNA was used as a template and Real time PCR was performed using KAPA SYBR Fast qPCR master mix (Sigma) according to manufacturer’s instructions in Step One Plus Real Time PCR system (Applied Biosystems). Primers for RT-PCR were designed using Primer 3 software (Table S4). Results were normalized to *sigA* as an endogenous control^13^.

### Oxygen consumption rate measurements

Mid-log phase cultures of *M*. *bovis* (BCG) were washed and inoculated at an initial OD_600_ of 0.2 in 7H9 liquid broth media supplemented with and without 0.2% glycerol. Bacterial cultures in their respective media were incubated at 37 °C for 48 hrs in presence or absence of Q203. Cells were harvested by centrifugation and re-suspended in their respective culture broth medium. The basal oxygen consumption rates (OCR) of *M*. *bovis* BCG were measured using the Seahorse XFe96 Analyzer (Agilent). 7 × 10^6^ cells were added to each well on the Agilent Seahorse cell culture microtiter plates coated with 22.4 µg/mL Cell Tak. The cells were adhered to the bottom of the plate by centrifugation. The OCR data points were derived from 4 minutes of continuous measurements. The representative basal OCR was an average of 6 measurements. The experiment concluded with an injection of CCCP (2 µM), followed by 5 additional measurement points. All data are expressed as the mean and error of triplicates, calculated using the Wave Desktop 2.2 software^43^.

## Supplementary information

Carbon metabolism modulates the efficacy of drugs targeting the cytochrome bc1:aa3bc111:aa333bc1:aa3 in Mycobacterium tuberculosis</b>Mycobacterium tuberculosis</b><i>Mycobacterium tuberculosis
