# Supplementary material for: Carbon metabolism modulates the efficacy of drugs targeting the cytochrome bc1:aa3 in Mycobacterium tuberculosis
Source: Sci Rep. 2019 Jun 13;9:8608. doi: 10.1038/s41598-019-44887-9 (PMC6565617; doi:10.1038/s41598-019-44887-9)
Supplement: Supplementary file 1 — Carbon metabolism modulates the efficacy of drugs targeting the cytochrome bc1:aa3bc111:aa333bc1:aa3 in Mycobacterium tuberculosisMycobacterium tuberculosisMycobacterium tuberculosis [file 41598_2019_44887_MOESM1_ESM.pdf]

Carbon metabolism modulates the efficacy of drugs targeting the cytochrome *bc_1:aa_3* in *Mycobacterium tuberculosis*

Nitin P. Kalia[1], Bei Shi Lee[2], Nurlilah B. Ab Rahman[1], Garrett C. Moraski,[3] Marvin J. Miller,[4] Kevin Pethe[1, 2*]

[1]Lee Kong Chian School of Medicine, Nanyang Technological University, Singapore 636921.

[2]School of Biological Sciences, Nanyang Technological University, Singapore 637551.

[3]Department of Chemistry and Biochemistry, Montana State University, Bozeman, MT, USA 59717.

[4]Department of Chemistry and Biochemistry, University of Notre Dame, Notre Dame, IN, USA 46556.

Methods

Glycerol Quantification: Briefly, cells from mid-log phase culture of *M. bovis* (BCG) were washed twice in 7H9 base medium supplemented with 0.05% tyloxapol, without carbon sources and OD_{600} was adjusted to 0.01 in 7H9 liquid broth media supplemented with 0.1 % fatty acid free BSA, 0.8% NaCl, and 0.2 % glycerol as a dominant carbon source. Extracellular glycerol was quantified in Q203 treated and untreated *M. bovis* over a 12 days period using Glycerol Assay Kit (Abcam, ab133130) according to manufacturer's instruction on BioTek Cytation multimode reader.

Figure Legends

Figure S1. Limited mycobacterial growth in the absence of a dominant carbon source. *M. bovis* (BCG) was grown in defined, home-made, 7H9-base liquid broth media supplemented with glycerol (red circles) as a sole carbon source and without supplementation with a dominant carbon source (green squares). Bacterial growth was monitored over a 10 days period. Data are expressed as the mean ± SDs.

Figure S2. Effect of carbon metabolism on the potency of Q203 in *M. bovis* BCG and *M. bovis* BCGΔ*cydAB*. The MIC_{50} of Q203 (A,C), and bedaquiline (B,D) were determined in liquid broth media with glycerol (red circles), glucose (green squares), pyruvate (blue triangles), acetate (purple diamonds) and propionate (pink hexagon) as sole carbon sources. After 10 days of incubation bacterial growth was recorded by measuring the optical density at 600 nm. Data are expressed as the mean ± SDs.

Figure S3. Q203 treatment slowed down, but did not inhibit glycerol consumption in mycobacteria. *M. bovis* (BCG) and was incubated in defined liquid broth media supplemented with glycerol as a sole dominant carbon source. Extracellular glycerol concentration was measured in Q203 treated (green squares) and untreated (red circles) *M. bovis* over a 12 days period (A). Glycerol concentration was measured in media without bacteria at day 0 and after 12 days of incubation (blue bars), in Q203-treated and untreated BCG (red bars) and in BCGΔ*cydAB* (green bars) after 12 days of incubation. Results are expressed as mean ± SDs.

Figure S4. Over-expression of *cydABDC* operon diminishes partially Q203-mediated growth inhibition on acetate-supplemented culture broth medium. *M. bovis* (BCG) (red circles) and BCG::pMV262*cydABDC* (green squares) were incubated in defined liquid broth media supplemented with acetate as sole dominant carbon source in the presence of 100 nM of Q203. Bacterial growth was monitored over a 20-day period. Error bars represent the standard deviation (SD) of three biological replicates.

Figure S5. Complementation of the H37RvΔ*cydAB* with a single *cydAB* copy restores the detrimental effect of glycerol supplementation on Q203 potency. *M. tuberculosis* H37RvΔ*cydAB* and Δ*cydAB*comp strains were exposed to a dose range of Q203 (A, C) or BDQ (B, D) in liquid media supplemented (red circles) or not (green squares) with glycerol. Bacterial growth was measured by recording the Optical Density at 600 nm after 5 day of incubation. Results are expressed as mean ± SD. Experiments were performed in triplicate and repeated once.

Fig. S1

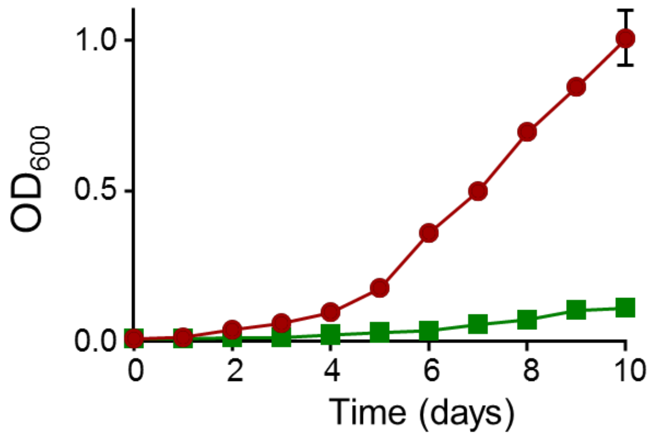

Fig. S2

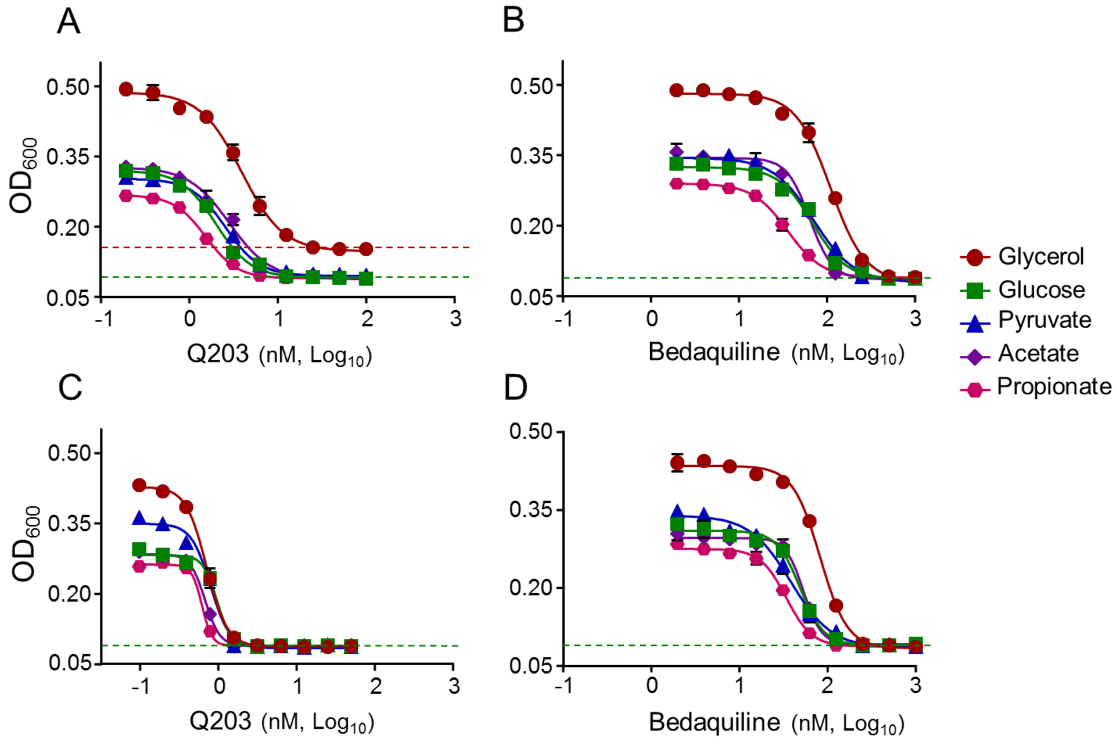

A
B
C
D
0.50
0.35
0.20
0.05
OD_{600}
Q203 (nM, Log_{10})
Bedaquiline (nM, Log_{10})
-1
0
1
2
3
Glycerol
Glucose
Pyruvate
Acetate
Propionate

Fig. S3

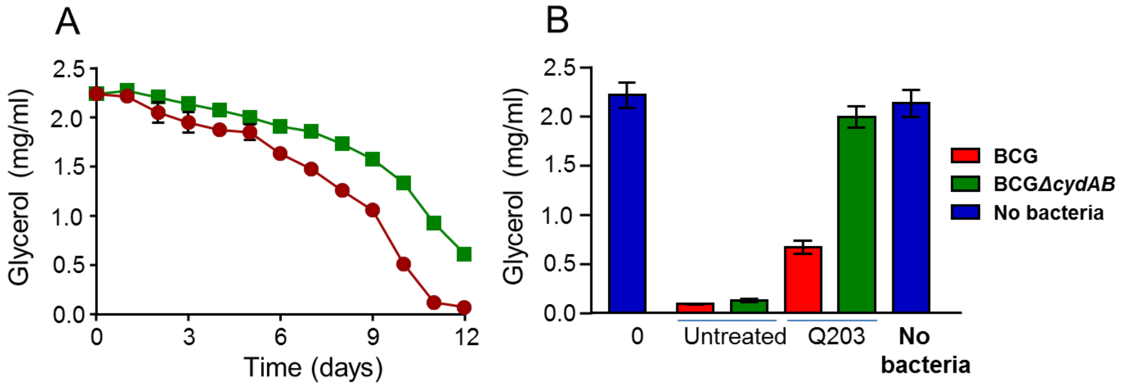
A
2.5
2.0
1.5
1.0
0.5
0.0
Glycerol (mg/ml)
0
3
6
9
12
Time (days)
B
2.5
2.0
1.5
1.0
0.5
0.0
Glycerol (mg/ml)
0
Untreated
Q203
No bacteria
BCG
BCGΔcydAB
No bacteria

Fig. S4

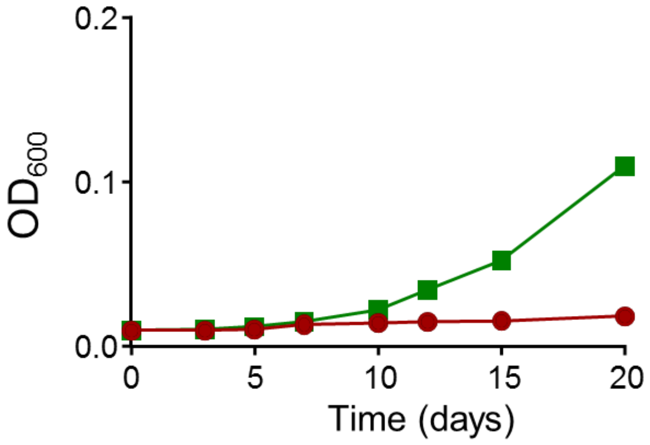

Fig. S5

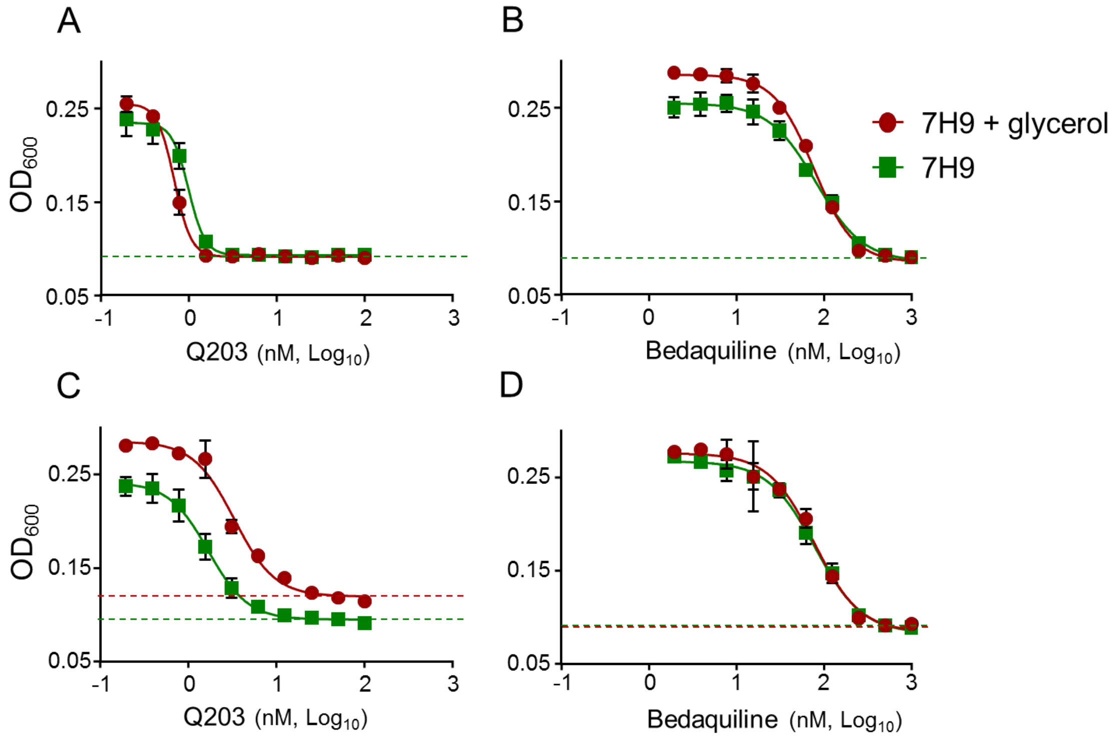
A
B
C
D
OD$_{600}$
0.25
0.15
0.05
-1
0
1
2
3
Q203 (nM, Log$_{10}$)
Bedaquiline (nM, Log$_{10}$)
7H9 + glycerol
7H9

Table S1. MIC_{50} of Q203 and BDQ against *M. tuberculosis* H37Rv and *M. bovis* (BCG) in liquid broth media with different sole carbon sources

	M. tuberculosis **H37Rv**		***M. bovis*** **(BCG)**	
	Q203 (MIC_{50}, nM)	BDQ (MIC_{50}, nM)	Q203 (MIC_{50}, nM)	BDQ (MIC_{50}, nM)
Glycerol	4.7 ± 0.7	116 ± 2.1	3.9 ± 0.6	110.4 ± 2.0
Glucose	4.0 ± 0.60	87.3 ± 1.9	3.0 ± 0.4	69.4 ± 1.8
Pyruvate	2.6 ± 0.4	42.5 ± 1.6	2.6 ± 0.4	70.8 ± 1.9
Acetate	2.3 ± 0.3	78.9 ± 1.9	2.8 ± 0.5	64.0 ± 1.8
Propionate	1.5 ± 0.15	32.3 ± 1.5	1.5 ± 0.17	35.9 ± 1.60

Table S2. MIC_{50} of Q203 and BDQ against *M. tuberculosis* H37RvΔ*cydAB* and *M. bovis* BCGΔ*cydAB* in liquid broth media with different sole dominant carbon sources

	**H37Rv Δ*cydAB***		**BCGΔ*cydAB***	
	Q203 (MIC_{50}, nM)	BDQ (MIC_{50}, nM)	Q203 (MIC_{50}, nM)	BDQ (MIC_{50}, nM)
Glycerol	0.8 ± 0.05	94.9 ± 1.9	0.7 ± 0.04	82.4 ± 1.9
Glucose	1.0 ± 0.03	63.8 ± 1.8	1.0 ± 0.01	48.9 ± 1.7
Pyruvate	0.7 ± 0.1	51.1 ± 1.7	0.8 ± 0.1	38.3 ± 1.6
Acetate	1.0 ± 0.03	68.9 ± 1.8	0.7 ± 0.2	53.4 ± 1.7
Propionate	0.7 ± 0.2	42.5 ± 1.6	0.6 ± 0.04	34.0 ± 1.5

Table S3. Mutation frequency of mutation and amino acid substitutions in *qcrB* associated with Q203

		Glycerol		Pyruvate	
	Q203 (nM)	Mutation frequency	QcrB Substitution (No of mutants with mutation/Total No. of mutants analyzed)	Mutation frequency	QcrB Substitution (No of mutants with mutation/Total No. of mutants analyzed)
BCG	500	Lawn	-	6.7×10^{-9}	ND*
	100	Lawn	-	1.0×10^{-8}	T313A (2/2)
BCG *ΔcydAB*	100	4.3×10^{-8}	T313A (2/2)	-	-
	25	5.0×10^{-8}	T313A (1/2), V338G (1/2)	-	-
	5	8.7×10^{-8}	T313A (3/7), V338G (4/7)	-	-

*ND: Not determined

Table S4. Primer sequences used for quantitative RT-PCR

**Gene name**	**Primer Sequence (5' to 3')**	
	Forward	Reverse
sigA	ATCTCGTTGGACCAGACCAT	TGCAGCAAAGTGAAGGACAC
cydA	CATAGGGCACTTCGATGACA	ACCAAGGCAAGCTGATGTTC
cydB	AATCGACAGGGCAACATGAC	GCGATCCTGTTCGGTATGAT
qcrC	CATTGTCGTCCGGCAAATAC	GTTGGAGAACTTCGGCATGT
qcrA	CACACCATAAGCTGCAGGAA	ATGAGAGCAGACCTTGGTGAAC
qcrB	GGGTAGTGGCAGTTTTCTGTTC	CATGATGCTGTCCTGGTGTT
